# Supplementary material for: Evaluation of the ActiMotus Software to Accurately Classify Postures and Movements in Children Aged 3–14
Source: Sensors (Basel). 2024 Oct 18;24(20):6705. doi: 10.3390/s24206705 (PMC11510827; doi:10.3390/s24206705)
Supplement: Supplementary file 1 [file sensors-24-06705-s001.zip › sensors-3236471-supplementary.pdf]

Supplementary File 1

Table S1. *Run sheet used during the data collection session.*

Device	Posture/movement	3-5 years	6-10 years	11-14 years
TV	Sitting (bean bag)	Watching ABC Kids programme sitting on bean bag	Watching ABC Kids programme sitting bean bag	Watching ABC ME programme sitting on bean bag
TV	Lying (prone)	Watching ABC Kids programme lying (prone) on floormat	Watching ABC Kids programme lying (prone) on floormat	Watching ABC ME programme lying (prone) on floormat
Laptop	Lying (side lying)	Watching ABC Kids programme lying (side) on floormat	Watching ABC Kids programme lying (side) on floormat	Watching ABC ME programme lying (side) on floormat
Laptop	Sitting (chair)	Playing ABC Kids game sitting on chair at the desk	Playing ABC Kids game sitting on chair at a desk	Playing Avatar Maker game sitting on chair at a desk
Handheld gaming	Sitting (couch)	Playing Mario Kart via Nintendo Switch sitting on couch	Playing Mario Kart via Nintendo Switch) sitting on couch	Playing Mario Kart via Nintendo Switch sitting on couch
Book reading	Lying (prone)	Reading a book lying (prone)	Reading a book lying (prone)	Reading a book lying (prone)
Magnetic Tiles	Sitting (floor)	Playing with magnetic tiles, sitting on the floor	Playing with magnetic tiles, sitting on the floor	Playing with magnetic tiles, sitting on the floor
Passive gaming	Sitting (couch)	Playing Mario Kart via Nintendo Switch with parent (optional) sitting on couch	Playing Mario Kart via Nintendo Switch with parent (optional) sitting on couch	Playing Mario Kart via Nintendo Switch with parent (optional) sitting on couch
Desktop	Sitting (chair)	Video calling via Microsoft Teams sitting on chair	Video calling via Microsoft Teams sitting on chair	Video calling via Microsoft Teams sitting on chair
Active gaming	Standing while moving +/- jumping	Playing Just Dance via Nintendo Switch whilst standing	Playing NS Sports/Just Dance via Nintendo Switch whilst standing	Playing NS Sports/Just Dance via Nintendo Switch whilst standing
Tablet	Standing	Playing Art Maker app whilst standing at a table	Playing Art Maker or Bitmoji app whilst standing at a table	Playing Bitmoji app whilst standing at a table

Drawing	Standing	Standing and recreate drawing on paper/colour in	Standing and recreate drawing on paper/colour in	Standing and recreate drawing on paper/colour in
Tablet	Standing whilst moving	Watching Cosmic Kids Yoga video whilst standing/moving	Watching Cosmic Kids Yoga video whilst standing/moving	Watching Down Dog Yoga app whilst standing/moving
Mobile	Walking; Jumping; Running; Skipping	Taking selfies/pictures of posters whilst walking/jumping/ running/skipping	Taking selfies/pictures of posters whilst walking/jumping/ running/skipping	Taking selfies/pictures of posters whilst walking/jumping/ running/skipping
Throwing/ Catching a ball	Standing with movement or walking variation	Throwing/catching a netball/ basketball	Throwing/catching a netball/ basketball	Throwing/catching a netball/ basketball
Mobile	Lying supine/ crook lie (floor)	Watching Smiling Minds Sea Creatures video whilst lying supine on floor	Watching Smiling Minds Sea Creatures video whilst lying on floor	Watching Smiling Minds Sea Creatures video whilst lying on floor
Smartwatch	Jumping; Handstands	Jumping and then checking heart rate/steps whilst standing	Jumping and then checking heart rate/steps whilst standing	Jumping and then checking heart rate/steps whilst standing
Smartwatch	Running; Cartwheels	Running and then checking heart rate/steps whilst standing	Jumping and then checking heart rate/steps whilst standing	Jumping and then checking heart rate/steps whilst standing
Smartwatch	Climbing stairs	Climbing stairs with or without adult	Climbing stairs independently	Climbing stairs independently
Free play	Child chooses activity they would like to do again			

All activities were age-appropriate and based on types of plays that the children might enjoy doing.
